# Climate Change and Elderly Americans: Examining Adaptability in an Aging Population

**DOI:** 10.1289/ehp.121-a33

**Published:** 2013-01-01

**Authors:** Tanya Tillett

**Affiliations:** Tanya Tillett, MA, of Durham, NC, is a staff writer/editor for *EHP*. She has been on the *EHP* staff since 2000 and has represented the journal at national and international conferences.

Adults aged 65 and older make up about 13% of the current U.S. population, but by 2040 that number is projected to increase to 20%. One of the most significant public health challenges facing this aging population will be changes in the frequency and/or intensity of climate-related stressors such as hot temperatures and extreme weather events. A new review of the literature provides a comprehensive assessment of the state of the science regarding the impacts of climate change on this segment of the U.S. population and discusses possible adaptation measures [*EHP* 121(1):15–22; Gamble et al.].

The investigators performed PubMed and Google Scholar literature searches for relevant research dating from 2000 through 2011 using three sets of general search terms describing 1) the older adult population; 2) environmental impacts, events, and potentially vulnerable areas related to climate change; and 3) effects of climate change on health and well being. In addition, they polled subject matter experts and reviewed major synthesis reports to identify additional relevant sources. Ultimately they created a list of nearly 100 papers and reports for analysis, including a few seminal articles published earlier than 2000.

The number of older adults in the United States is projected to increase to 88.5 million by the year 2050, with 21% of these individuals aged 85 or older. Relatively higher concentrations of older adults live in areas such as coastal zones, large urban areas in the Northeast and Midwest, and in the Southwest, all of which are expected to be especially affected by climate stressors such as heat events, droughts, and wildfires; hurricanes, storm surges, floods, and sea-level rise; and higher concentrations of ground-level ozone and other air pollutants and airborne allergens.

**Figure:**
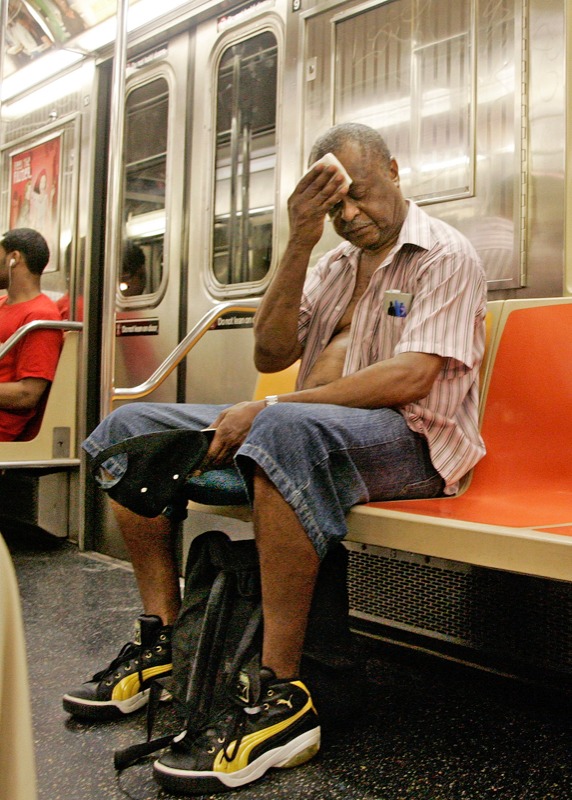
New York City, 2 August 2006. Back-to-back heatwaves in the summer of 2006 killed dozens of New Yorkers, many of them over age 65. © AP Photo/Richard Drew

Socioeconomic characteristics are expected to contribute to older adults’ vulnerability to climate-related stressors. Elderly adults living in poverty or on limited fixed incomes may lack resources to pay for air conditioning during heat waves, they may live in substandard housing that leaves them more vulnerable to flooding and strong winds, or they may not have easy access to social services or to adequate transportation to evacuate when devastating weather events occur. In addition, functional and physiological limitations associated with aging could impede elderly people’s ability to adapt.

Implementing effective adaptation measures will be key to addressing the unique risks faced by the elderly in association with projected changes in climate. The authors discuss a number of resources that could aid in this effort, such as community-based registries of at-risk older adults, local outreach programs targeted toward older community members, and the development of preemptive ecological strategies designed to temper heat and promote green environments, for instance, planting shade trees and other vegetation and installing green roofs. But more research is needed to assess the effectiveness of adaptation measures and to identify and implement those that are most useful.

